# Combined effects of photobiomodulation and osteopathic manipulation on the physical performance of young adults subjected to exercise-induced muscle damage: a randomized placebo-controlled trial

**DOI:** 10.1007/s10103-026-04907-3

**Published:** 2026-06-11

**Authors:** Joyce Cristina Freitas, Mayara Bocchi, Benilton Alves Rodrigues Junior, Monica Rodrigues Ferreira Machado, Hanstter Hallison Alves Rezende, David Michel de Oliveira, Eduardo Vignoto Fernandes

**Affiliations:** 1 Laboratory of Immunometabolism, Nutrition and Exercise, Federal University of Jataí, Jataí, Brazil; 2 Laboratory of Bacteriology and Mycology, Federal University of Jataí, Jataí, Brazil; 3 Postgraduate Program in Bioscience and One Health, Federal University of Jataí, Jataí, Brazil

**Keywords:** Muscle injury, High-velocity low-amplitude thrust, Post-exercise recovery, Physical functional performance

## Abstract

The aim of this study was to investigate the combined effects of photobiomodulation (PBM) and osteopathic manipulation (OM) on the physical performance of young adults subjected to exercise-induced muscle damage (EIMD). The study is a randomized, placebo-controlled clinical trial. Forty-five physically active male individuals were included and randomized into four groups: placebo (PL), PBM, OM, and PBM + OM. A red-light emitting diode (LED) (λ = 650 nm) was used. Participants in the PBM and PBM + OM groups received 144 J of energy in the right lower limb and/or underwent bilateral lumbar and pelvic manipulation (OM and PBM + OM) before the EIMD protocol. The following variables were analyzed before, immediately after, and 24, 48, and 72 h post-intervention: creatine kinase (CK), muscle strength, flexibility, and horizontal jump performance. Data normality was assessed using the Kolmogorov-Smirnov test. CK levels were evaluated using the Friedman test, and repeated measures ANOVA was used for the other variables, *p* < 0.05. A distinct recovery pattern was observed in the combined PBM + OM group, with increased CK levels only at 24 h, followed by a pronounced reduction at 72 h (*p* = 0.001). Only the PBM + OM group presented percentage values near to baseline within 48 h (-1.4%), in addition to a trend toward early improvement in the horizontal jump within 24 h. In conclusion, PBM + OM may have contributed to improved CK kinetics and selected functional outcomes; however, strength recovery should be interpreted strictly as a non-significant and exploratory trend.

## Introduction

Exercise-induced muscle damage (EIMD) occurs after prolonged or intense sessions of physical exercise (PE), and primarily affects individuals unaccustomed to the specific exercise modality [1]. High-intensity exercise associated with mechanical and metabolic stress is a contributing factor to EIMD [2]. Among the various types of exercise commonly practiced, those involving high-intensity eccentric contractions, such as plyometric exercise, are prominent models for inducing damage to sarcomere structures. This damage is often accompanied by an intramuscular inflammatory response and symptoms associated with EIMD, which should be distinguished from transient muscle fatigue [3]. This damage is characterized by inflammatory processes, reduced muscle function, and increased serum levels of creatine kinase (CK), and typically peaks between 24 and 72 h after PE [4]. Some studies have explored the ability of passive (e.g., cryotherapy and massage) and active (e.g., low-intensity aerobic exercise) recovery strategies to minimize the adverse effects of EIMD and optimize muscle recovery, including photobiomodulation (PBM) and osteopathic manipulation (OM) [1, 5–8].

Due to its potential anti-inflammatory and regenerative effects, and ability to reduce biomarkers of muscle injury, such as CK, PBM is commonly used in the treatment of musculoskeletal injuries [9]. The literature demonstrates the positive influence of PBM in reducing inflammation and muscle fatigue, while improving muscle strength and the rate of tissue adaptation [10]. The mechanism of action is related to an increase in the cytochrome c oxidase enzyme in skeletal muscle fibers, which accelerates the flow of electrons in the respiratory chain, and generates greater pumping of protons into the intermembrane space, enhancing the electrochemical gradient. This results in increased production of adenosine triphosphate (ATP) and more energy for the muscle, while simultaneously reducing oxidative stress and the production of reactive oxygen species [11]. Consequently, this enhanced energy availability and the reduced oxidative damage translate into superior functional capacity, sustaining physical performance even under conditions of exercise-induced stress [10, 12].

Osteopathic manipulation (OM) consists of manual techniques applied to different structures of the body. Among the techniques used, high-velocity, low-amplitude (HVLA) maneuvers have demonstrated effectiveness in improving neuromuscular function and motor control [13]. Studies indicate that OM applied to the pelvis can result in an immediate increase in isometric strength of the lower limbs in healthy individuals, including in conditions following fatigue or sports activities [14], and that spinal mobilization and manipulation maneuvers can improve spinal and hip mobility [15]. While not directly measured in the present study, literature suggests that the potential mechanisms underlying these findings may involve the activation of proprioceptive afferent neurons, including muscle spindles and Golgi tendon organs, which are sensitive to rapid changes in muscle length and tension [16]. Theoretically, these sensory afferents could influence the central nervous system (CNS), potentially modulating motor neuron excitability and recruitment. From a functional perspective, this hypothesized neural optimization could play a role in restoring athletic maneuvers, such as vertical and horizontal jump capacity, following exercise-induced impairment [14, 17, 18]. However, as neurophysiological markers were not assessed in our protocol, these mechanistic pathways remain speculative and warrant further direct investigation.

It is known that EIMD is a common phenomenon among physical exercise practitioners, often resulting in reduced muscle strength and physical capacity, and limited range of motion [4]. Although several well-known strategies, such as medication, cryotherapy, and active recovery are commonly used to minimize EIMD, they often present side effects or limited benefits. In this scenario, PBM and OM emerge as safe and physiologically complementary alternatives; while PBM targets cellular recovery and oxidative stress, OM addresses neuromuscular inhibition and biomechanical alignment, suggesting a potential complementary benefit to optimize tissue recovery and functional performance [10, 13]. However, no studies were found in the literature that verified the combined application of these two interventions. In this context, the present study aims to investigate the combined effects of PBM and OM on the physical performance of young adults undergoing EIMD. We hypothesized that the combination of PBM and OM would promote an accelerated recovery profile, resulting in a faster reduction in biochemical markers of muscle damage (CK) and earlier recovery of physical performance (strength, flexibility, and jump capacity) compared to isolated interventions or the placebo.

## Materials and methods

### Type of study, location and participants

The current study is a randomized, placebo-controlled clinical trial conducted at the Laboratory of Immunometabolism, Nutrition and Exercise of the Federal University of Jataí (UFJ), Jataí, Goiás, Brazil.

The study was conducted in accordance with the Declaration of Helsinki, approved by the Institutional Human Research Ethics Committee of UFJ (opinion: 6.885.282; CAAE: 79824024.3.0000.0187), and registered on the Brazilian Registry of Clinical Trials (ReBEC) platform under number RBR-8kdv3pt (https://ensaiosclinicos.gov.br/rg/RBR-8kdv3pt*)*, registered on July 15, 2024. Prior to formal inclusion, all participants were comprehensively briefed on the study’s experimental design. The written informed consent process explicitly detailed all involved procedures, including invasive blood sampling, High-Velocity Low-Amplitude (HVLA) manual osteopathic manipulations, photobiomodulation application, and the eccentric exercise-induced muscle damage protocol. Furthermore, volunteers were fully informed about potential risks and physical discomforts—such as temporary delayed-onset muscle soreness or minor local tenderness—the monitoring and management of potential adverse events, and their unconditional right to withdraw from the study at any time without any form of penalty or loss of benefits. All participants provided written informed consent before the initiation of any experimental procedures.

The sample size was calculated a priori using G*Power software, version 3.1.9.4 (Institute for Experimental Psychology, Düsseldorf, Germany), based on a two-way mixed-effects model (Repeated Measures ANOVA/REML) for within-between interactions (4 × 5 time points). The calculation was based on the kinetics of creatine kinase (CK) recovery as the primary outcome, derived from previous literature [3], which demonstrated a 100% recovery of CK after 72 h in photobiomodulation groups compared to an 85% recovery in the placebo group. Assuming a large, expected effect size of f = 0.40 (η_p_² = 0.14), an α level of 0.05, a statistical power (1- β) of 0.80, and an allocation ratio of 1:1:1:1, the minimum required sample size was determined to be 32 participants (8 per group).

**Fig. 1:**
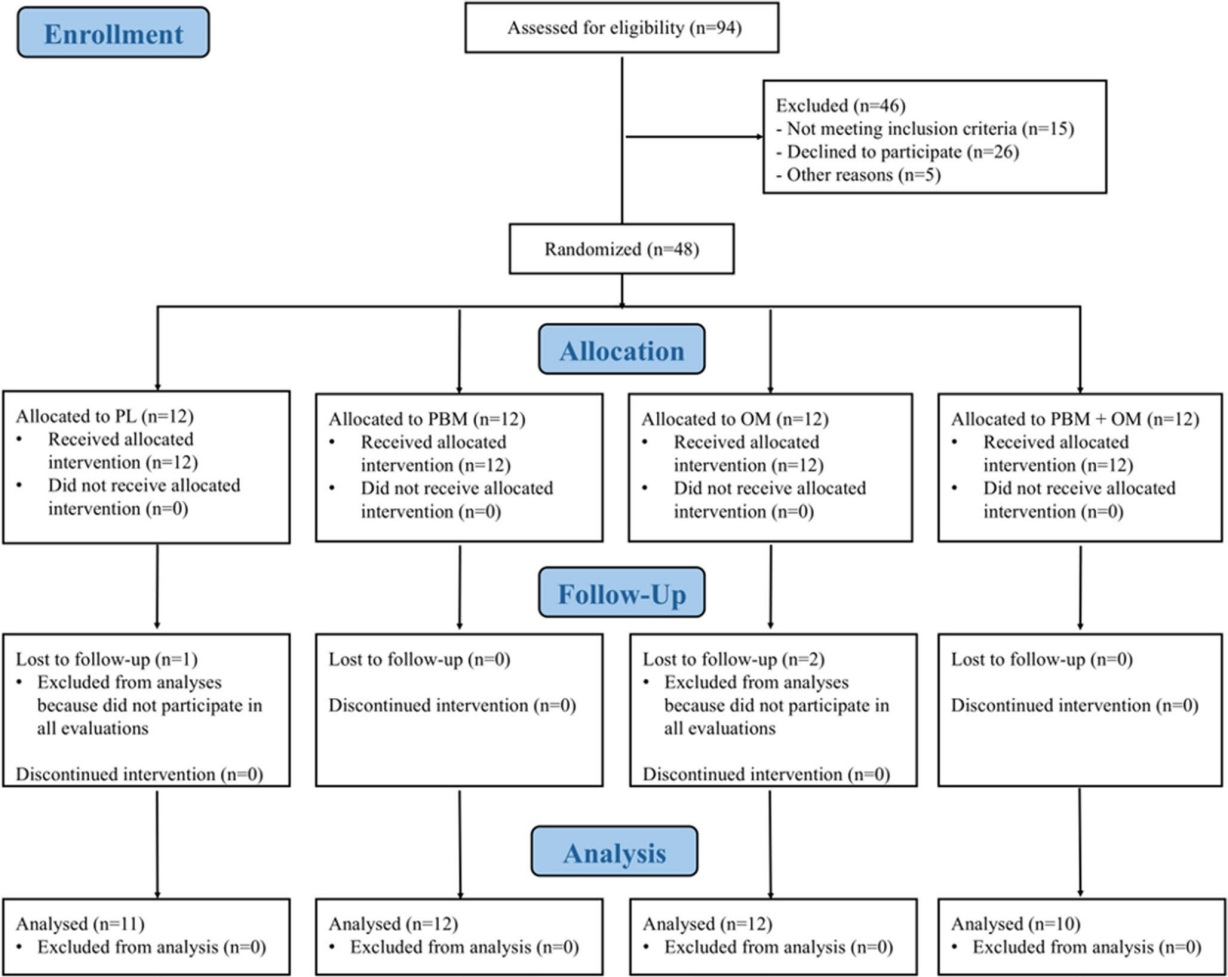
Study flowchart. PL: Placebo; PBM: Photobiomodulation; Photobiomodulation + Osteopathic Manipulation; OM: Osteopathic Manipulation

Participants were recruited through social media posts and screened via Google Forms^®^. In total, 94 individuals responded to the questionnaire, of whom 48 met the inclusion criteria and were randomized into four groups (12 per group): Placebo (PL), Photobiomodulation (PBM), Osteopathic Manipulation (OM), and PBM + OM (Fig. 1). While all 48 randomized participants completed the biochemical follow-up (providing 100% of data for CK analysis), 3 participants were excluded from the final mechanical analysis because they failed to participate in all stages of the study due to logistical non-compliance during the post-exercise functional evaluation blocks. Consequently, 45 participants completed the physical performance outcomes (strength, flexibility, and horizontal jump), which still strictly satisfies and exceeds the minimum power requirements of 32 participants established by the a priori sample size calculation.

### Inclusion and exclusion criteria

Inclusion criteria: participants were required to be male and between 18 and 35 years of age. In addition, subjects were required to have at least 6 months of experience in resistance and aerobic training, with at least 150 min of moderate to intense physical activity per week, according to the recommendations of the Canadian Physical Activity Guideline for adults [19]. Furthermore, for inclusion, participants could not have engaged in plyometric or jumping-based activities—such as volleyball, basketball, or high-intensity functional training (CrossFit)—within the preceding six months. This restriction ensured that participants were unaccustomed to loading patterns and exercise protocols that could induce neuromuscular adaptations prior to the drop jump test protocol.

Exclusion criteria: use of analgesic/anti-inflammatory supplements or medications in the seven days prior to and during data collection; consumption of alcohol or ergogenic substances in the 72 h preceding the tests; musculoskeletal injuries up to six months before the study and during data collection; and failure to participate in all stages of the study [3].

### Randomization and blinding

The randomization process involved generating numbers using a random number table (www.random.org) and concealing the allocation with opaque, sealed envelopes. The group assignment for each participant was provided to an independent researcher who was not involved in the interventions, data collection, or statistical analysis. Consequently, the principal investigator (who conducted the statistical analysis) and the outcome evaluators remained strictly blinded to group allocation. Due to the nature of the manual therapy interventions, the therapist performing the techniques could not be blinded to the OM assignments; however, they remained blinded to the participants’ baseline data and subsequent clinical outcomes.

To ensure participants remained blinded, all individuals underwent all protocol stages. All procedures were performed in a private room, with only the participant and the evaluator present. During the PBM application, all participants were blindfolded and wore headphones with music to ensure blindness. For the OM and PL groups, a sham PBM procedure was performed with the active equipment turned off. During the sham OM (for the PBM and PL groups), participants were placed in the exact same clinical positions as those receiving the active intervention (OM and PBM + OM), and the therapist simulated the hand placement without delivering any therapeutic thrust or maneuver.

### Experimental design

Initially, anthropometric information, body composition (bioimpedance), blood type, physical activity level, and physical capacity (isometric quadriceps muscle strength, horizontal jump, and flexibility) were collected. After the initial data collection, participants underwent interventions with PBM, OM, or PBM + OM. The PL group underwent the respective sham protocols (sham PBM and sham OM) to control for placebo effects.

**Fig. 2:**
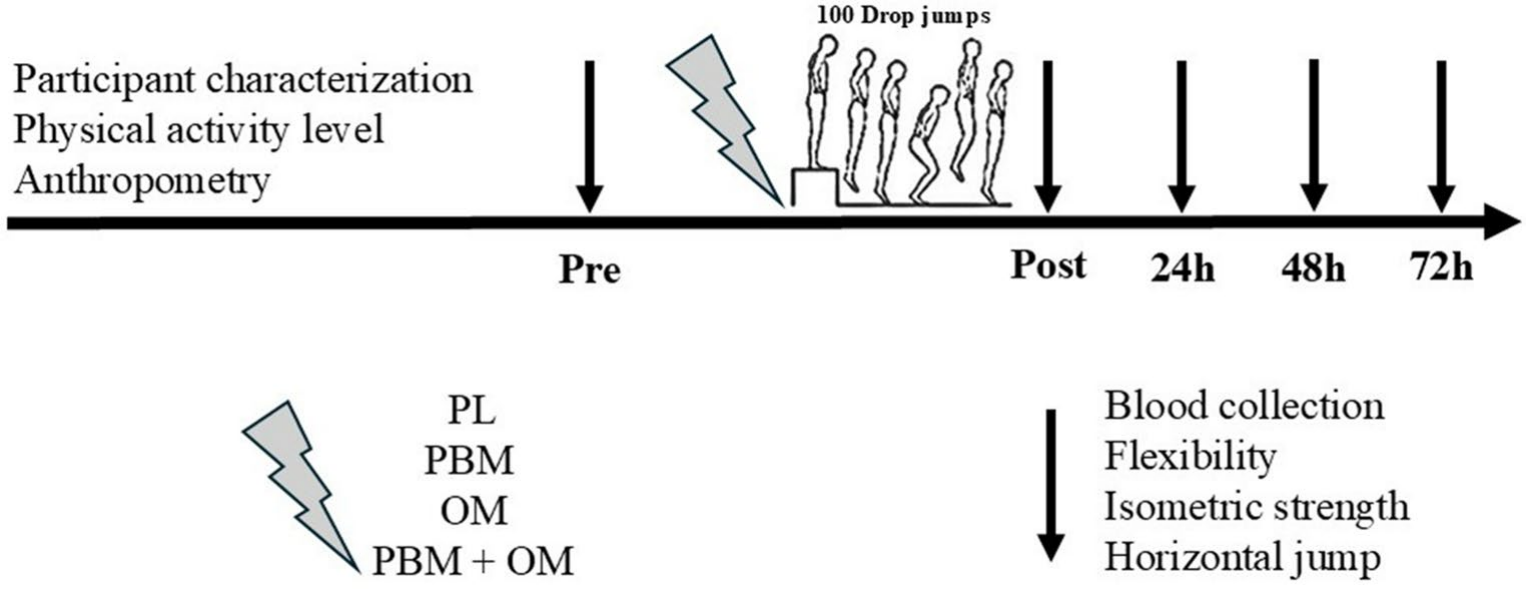
Dynamics of the timeline of data collection and interventions. h: hours; PL: Placebo; PBM: Photobiomodulation; PBM + OM: Photobiomodulation + Osteopathic Manipulation; OM: Osteopathic Manipulation

After the photobiomodulation intervention, participants performed the jump protocol, which consisted of 100 drop jumps [3]. Blood samples, isometric strength, and horizontal jump and flexibility tests were reapplied immediately after, and 24, 48, and 72 h after the intervention, Fig. 2.

### Procedures

#### Anthropometry and body composition

Body mass and composition were assessed using the Tanita^®^ BC-568 InnerScan bioimpedance scale (Tanita Corporation, Tokyo, Japan), which provided percentages of muscle mass, total fat, visceral fat, bone mass, body water percentage, basal metabolic rate, and metabolic age.

Height was measured using a Sanny^®^ ES2060 portable stadiometer (American Medical do Brasil Ltda, São Bernardo do Campo, Brazil) with a 1 mm precision. The body mass index (BMI) was calculated by dividing body mass (kg) by height in meters squared.

#### Blood collection and biochemical analyses

Blood collection was performed by trained biomedical professionals via venipuncture using the Vacutainer^®^ vacuum collection system, to obtain a 20 mL sample from each participant [20]. Samples were stored in tubes containing separator gel to obtain serum, and in tubes containing EDTA to obtain whole blood. Next, the samples were transported in insulated containers to the Laboratory of Bacteriology and Mycology, where they were centrifuged and the resulting serum was stored at −20 °C until the biochemical analysis.

Biochemical analysis was performed using the Sinnowa SX-3000^®^ semi-automatic analyzer (Sinnowa Medical Science & Technology Co., Ltd., Nanjing, Jiangsu, China). Concentrations of aspartate aminotransferase (AST), alanine aminotransferase (ALT), urea, creatinine, and total CK were determined using commercial reagents from Biotécnica^®^ (Varginha, MG, Brazil); total cholesterol, HDL cholesterol, and triglycerides were measured using reagents from Labtest^®^ (Lagoa Santa, MG, Brazil); and blood glucose was measured using a commercial kit from Wiener Lab^®^ (Rosario, Argentina). All procedures were conducted in accordance with the methodologies recommended by the respective manufacturers, as specified on the package inserts and certificates of analysis.

For hematological analysis, the Sysmex^®^ KX-21 N automated analyzer was used, with differential analysis of blood cells. A blood smear was prepared and subsequently stained for morphological analysis [21].

It is worth highlighting that the baseline assessments of the biochemical and hematological profiles were used to confirm the participants’ health status, and the CK assessment was used throughout the research to evaluate the biological effects of the intervention.

#### Flexibility

To assess the flexibility of the quadriceps femoris muscle of the right leg, the Ely Test was performed, with the participant in the prone position, knees supported on the examination table and ankles hanging off the Table [22]. The Sanny^®^ FL6010 fleximeter (American Medical do Brasil Ltda, São Bernardo do Campo, Brazil), with a scale of 0º to 360º, was placed with the dial facing the evaluator, on the lateral aspect of the ankle [23]. The participant performed three trials, and the maximum angular displacement was recorded.

#### Assessment of maximum isometric muscle power and strength

To measure the horizontal jump distance, a 10 m long, 25 mm wide tape measure (Profield^®^, São Paulo, Brazil) was used, fixed to the ground. A starting line was created at the zero point of the tape measure. The participants positioned themselves with their feet shoulder-width apart, arms relaxed at their sides, and the tape measure between their feet. They were instructed to perform a countermovement (flexing the knees and hips), to prepare for the jump. The participant performed a horizontal jump, as far as possible, propelling themselves forcefully with their lower limbs and using their arms to assist in propulsion. The jump distance was measured from the starting line to the heel closest to the landing mark [24]. Each participant made three jump attempts, and the highest score among the three was considered for the study.

The maximum isometric muscle strength of the quadriceps muscle was assessed using the SP Tech device (Medeor Medtech^®^, Florianópolis, SC, Brazil), a digital hand-held dynamometer that is able to record force up to approximately 90.72 kgf (200 lbf), operating with high-sensitivity load cell technology. Three repetitions were performed (contraction time – 5 s, relaxation time – 10 s). Reference position: seated in the leg extension machine with stabilizing belts (hip and distal thigh region), knee flexed at 90º. The lateral femoral condyle was used as the axis and aligned with the dynamometer axis in the seated position. To maintain the participant’s knee at 90º during the evaluation, a strap was placed around the ankle (3 cm above the lateral malleolus) and attached to the base of the leg extension machine [25].

#### Photobiomodulation (PBM)

PBM was performed using a Bios Therapy II device (Bios Indústria e Comércio de Equipamentos Médicos LTDA^®^, São José dos Campos, Brazil) with a 650 nm wavelength, positioned in contact with the skin over 16 points on the right lower limb, with eight points on the anterior thigh region (3 points on the rectus femoris, 3 on the vastus lateralis, and 2 on the vastus medialis), four points on the posterior thigh region (hamstring muscles), and four points on the posterior leg region (triceps surae). PBM was applied unilaterally to line up strictly with the specific dominant limb subjected to the localized drop jump test and subsequent mechanical evaluations.

All participants were positioned in the supine position for application of PBM to the anterior thigh region, and in the prone position for access to the posterior thigh and leg regions. The photobiomodulation protocol was performed as proposed by Padoin et al. [3]. Table 1 presents the treatment specifications.

**Table 1:** Photobiomodulation dosimetry

Parameters	
Wavelength	650 nm
Output frequency	Continuous
Output power	300 mW
Spot size area	1.3 cm²
Power density	230 mW/cm²
Number of points	16
Dose per point	9 J
Total energy per limb	144 J
Irradiated area	20.8 cm²
Energy density per point	6.9 J/cm²
Irradiation time per point	30 s
Total irradiation times	480 s
Application mode	Stationary, in skin contact

*cm*²: square centimeters, *J* Joules, *J/cm*² Joules per square centimeter, *mW* milliwatts, *mW/cm*² milliwatts per square centimeter, *nm* nanometers, *s* seconds

#### Osteopathic Manipulation (OM)

The manipulation techniques consisted of High-Velocity Low-Amplitude (HVLA) thrusts, applied by an experienced osteopath with over 5 years of clinical experience. Because of the nature of manual therapy, this investigator was unblinded to the group assignments. Two specific techniques were selected to address regional biomechanics and autonomic pathways: the lumbar roll for the upper lumbar region and a global pelvic manipulation. Both techniques were administered bilaterally because spinal and pelvic manual adjustments inherently target central biomechanical symmetry and autonomic nervous system tone, which cannot be restricted to a single side without compromising the clinical nature of the osteopathic approach. This bilateral framework differs from the unilateral PBM application, which was targeted strictly to the specific dominant limb subjected to the localized mechanical testing. Both techniques were performed as a single-session intervention, immediately before the exercise-induced muscle damage protocol. The clinical guidelines and positioning for these techniques followed the protocols described by Ricard [26].

To ensure adequate control for expectancy and placebo effects, participants allocated to the Placebo (PL) group underwent a strict, time-matched sham manipulation protocol by the same osteopath. Volunteers were placed in identical treatment positions (lateral decubitus and supine) for the exact same duration and experienced the same tactile contact and verbal interaction. However, the therapist deliberately withheld the final therapeutic high-velocity thrusts and mechanical work, executing only passive setup positionings without actual joint cavitation or corrective forces.

Exercise-induced muscle damage protocol (EIMD).

The EIMD was performed through five sets of 20 drop jumps, with 2-minute intervals between sets, on a platform elevated 50 cm from the ground. Participants were instructed to climb onto the platform and jump with their hands on their waists. Upon landing with both feet touching the ground simultaneously, they were instructed to bend their knees to approximately 90° and jump vertically as high as possible. With each jump, they alternated legs to climb the platform steps [3].

## Data analysis

Initially, data normality was assessed using the Kolmogorov-Smirnov test. To account for the 4 × 5 factorial design (Group times X Time), data were analyzed using a two-way mixed-effects model based on the restricted maximum likelihood approach. This framework was selected to evaluate the main effects of Group, Time, and the Group X Time interaction, while appropriately accommodating repeated measures and missing data points within the matrix without listwise deletion. Greenhouse-Geisser corrections were applied to account for violations of sphericity. Following the global model, within-group changes over time were further evaluated using repeated measures ANOVA (for parametric data) or the Friedman test (for non-parametric data), followed by Tukey’s or Dunn’s post-hoc tests, respectively.

To determine the practical magnitude of the within-group findings, effect sizes were calculated using Kendall’s coefficient of agreement (*W*) for non-parametric data and partial eta-squared (η_p_²) for parametric data. According to Cohen’s benchmarks for F-tests, η_p_² values were interpreted as small (0.01), medium (0.06), and large (≥ 0.14). Correlations between CK and functional variables (strength, flexibility, and horizontal jump) were performed using the Spearman rank correlation test. All statistical analyses were conducted using GraphPad Prism software (version 9.5.1). Results are expressed as mean±standard error of the mean (SEM), with the significance level set at *p* < 0.05.

## Results

### Participant profile and baseline characteristics

This subsection describes the demographic, anthropometric, and body composition characteristics of the participants across the four experimental groups. The data presented confirm the homogeneity of the sample and the effectiveness of the randomization process before any intervention (Table 2).

**Table 2:** Age, anthropometry, and body composition of the participants

Variables	PL	PBM	OM	PBM + OM	*P*
Age (years)	21.2 ± 3.8	21.7 ± 3.1	20.3 ± 4.0	21.3 ± 4.0	0.49†
Height (m)	1.76 ± 0.02	1.79 ± 0.02	1.75 ± 0.01	1.73 ± 0.01	0.42†
Body mass (kg)	83.72 ± 5.4	78.00 ± 4.3	72.68 ± 3.4	70.89 ± 3.9	0.15†
BMI (kg/m^2^)	26.83 ± 1.8	24.27 ± 1.4	23.68 ± 1.02	23.51 ± 1.4	0.39†
Muscle mass (%)	61.46 ± 2.1	61.18 ± 2.2	57.87 ± 2.4	55.71 ± 2.2	0.12†
Body fat (%)	20.88 ± 2.8	16.70 ± 1.5	16.22 ± 1.6	16.39 ± 1.7	0.53†
Visceral fat (%)	5.8 ± 1.6	3.1 ± 0.8	2.7 ± 0.5	3.3 ± 0.9	0.29†
Bone mass (%)	3.2 ± 0.1	3.2 ± 0.1	3.0 ± 0.1	2.9 ± 0.1	0.11†
Total body water (%)	60.00 ± 4.1	59.74 ± 1.3	60.88 ± 1.3	61.58 ± 1.7	0.92†
BMR (kcal)	1952 ± 73.7	1924 ± 76.2	1819 ± 75.9	1757 ± 69.1	0.13†
Metabolic age (years)	35.9 ± 7.3	21.3 ± 3.5	20.0 ± 2.5	22.8 ± 4.9	0.43†

*%* percentage, *kg/m*² BMI - Body Mass Index, *BMR* Basal Metabolic Rate, *kcal* kilocalories, *kg* kilograms, *kg/m*² kilograms per square meter, *m* meters, *OM* Osteopathic Manipulation, *PBM * photobiomodulation, *PBM + OM* Photobiomodulation + Osteopathic Manipulation, *PL* placebo. †One-way ANOVA statistical tests, data presented as mean ± standard error and minimum significance level of *p* < 0.05

The statistical analysis revealed no significant differences between the Placebo, PBM, OM, and PBM + OM groups for any of the baseline variables (*p* > 0.05). Specifically, the groups were balanced regarding age (*p* = 0.49), height (*p* = 0.42), body mass (*p* = 0.15), and BMI (*p* = 0.39). Furthermore, no differences were found in body composition markers, including muscle mass, body fat, bone mass, and total body water, as well as metabolic parameters such as BMR and metabolic age. These findings confirm that the participants shared a similar physical and physiological profile at the start of the study, ensuring that the subsequent results were not biased by baseline differences (Table 2).

### Baseline biochemical and hematological profile

This subsection presents the systemic health status of the participants before the experimental protocol, covering hepatic, renal, lipid, and glycemic markers, as well as full blood count. These reports demonstrate that all participants were within clinical reference ranges and no biochemical imbalances existed between groups at baseline (Table 3).

**Table 3:** Biochemical and hematological profile of the participants

Variables	PL	PBM	OM	PBM + OM	*P*	Reference values
AST (u/l)	27.6 ± 5.1	30.0 ± 12.1	17.6 ± 1.4	21.7 ± 4.1	0.22†	< 35 u/l
ALT (u/l)	29.0 ± 2.5	23.5 ± 6.4	17.8 ± 3	21.1 ± 5.1	0.37†	< 42 u/l
Urea (mg/dl)	30.8 ± 3.3	39.8 ± 2.4	47.7 ± 2.9	38.5 ± 5.9	0.07†	13–45 mg/dl
Creatinine (mg/dl)	1.0 ± 0.08	1.7 ± 0.8	0.8 ± 0.03	0.9 ± 0.04	0.46†	0.9–1.3 mg/dl
Triglycerides (mg/dl)	141.0 ± 18	83.9 ± 11.6	55.4 ± 9.1	101.8 ± 37.6	0.14†	< 150 mg/dl
Total cholesterol (mg/dl)	130.8 ± 9.6	130.3 ± 7.02	124.1 ± 6.6	125.0 ± 12.7	0.93†	< 200 mg/dl
HDL (mg/dl)	59.9 ± 3.7	58.6 ± 2.1	66.2 ± 4.1	55.8 ± 3.1	0.19†	30–70 mg/dl
Glucose (mg/dl)	87.0 ± 2.3	98.3 ± 3.3	90.8 ± 2.9	81.7 ± 3.9	0.23†	74–106 mg/dl
Hemoglobin (g/dl)	14.4 ± 0.1	14.8 ± 0.3	14.9 ± 0.2	14.9 ± 0.2	0.37†	13.5–17.5 g/dl
Hematocrit (%)	43.6 ± 0.4	44.7 ± 0.9	43.8 ± 0.8	45.0 ± 0.7	0.31†	41–53%
White Blood Cells (µl)	7.991 ± 0.5	7.650 ± 0.7	6.871 ± 0.6	9.924 ± 0.4	0.11†	4.000–11.000/µl
Neutrophils (%)	53.4 ± 1.3	53.2 ± 3.2	53.7 ± 2.1	53.2 ± 2.02	0.99†	40% – 70%
Lymphocytes (%)	29.9 ± 1.7	31.6 ± 3.3	30.7 ± 2.1	27.9 ± 2.7	0.78†	20% – 45%
Platelets (10^− 3^/µl)	269.5 ± 13.9	268.2 ± 28.1	242.8 ± 25.06	204.4 ± 19.8	0.11†	150–450 10^− 3^/µl
CK (u/l)	64.9 ± 54.9	76.3 ± 17.5	49.1 ± 25.8	53.1 ± 38.1	0.30‡	< 190 u/l

*%* percentage, *10*^− 3^/*µl* times ten to the power of three per microliter, *ALT* Alanine Aminotransferase, *AST* Aspartate Aminotransferase, *CK* Creatine Kinase, *g/dl* grams per deciliter, *HDL* High-Density Lipoprotein, *kcal* kilocalories, *kg* kilograms, *kg/m*² kilograms per square meter, *m* meters, *mg/dl* milligrams per deciliter, *OM* Osteopathic Manipulation, *PBM* Photobiomodulation, *PBM + OM* Photobiomodulation + Osteopathic Manipulation, *PL* Placebo, *u/l* units per liter, *µl* microliter. Statistical tests: †one-way ANOVA for parametric data and ‡Kruskal-Wallis for non-parametric data; data presented as mean ± standard error and minimum significance level of *p* < 0.05

The data analysis showed that results for all participants were within the clinical reference values for all variables analyzed. Furthermore, no statistically significant differences were observed between the PL, PBM, OM, and PBM + OM groups for any of the markers, including liver enzymes (AST, *p* = 0.22; ALT, *p* = 0.37), renal function (Urea, *p* = 0.07; Creatinine, *p* = 0.46), and inflammatory/muscle markers (White Blood Cells, *p* = 0.11; CK, *p* = 0.30). These findings confirm that the participants’ physiological profiles were balanced across all experimental conditions prior to the induction of muscle damage.

### Muscle damage markers and physical performance recovery

**Fig. 3:**
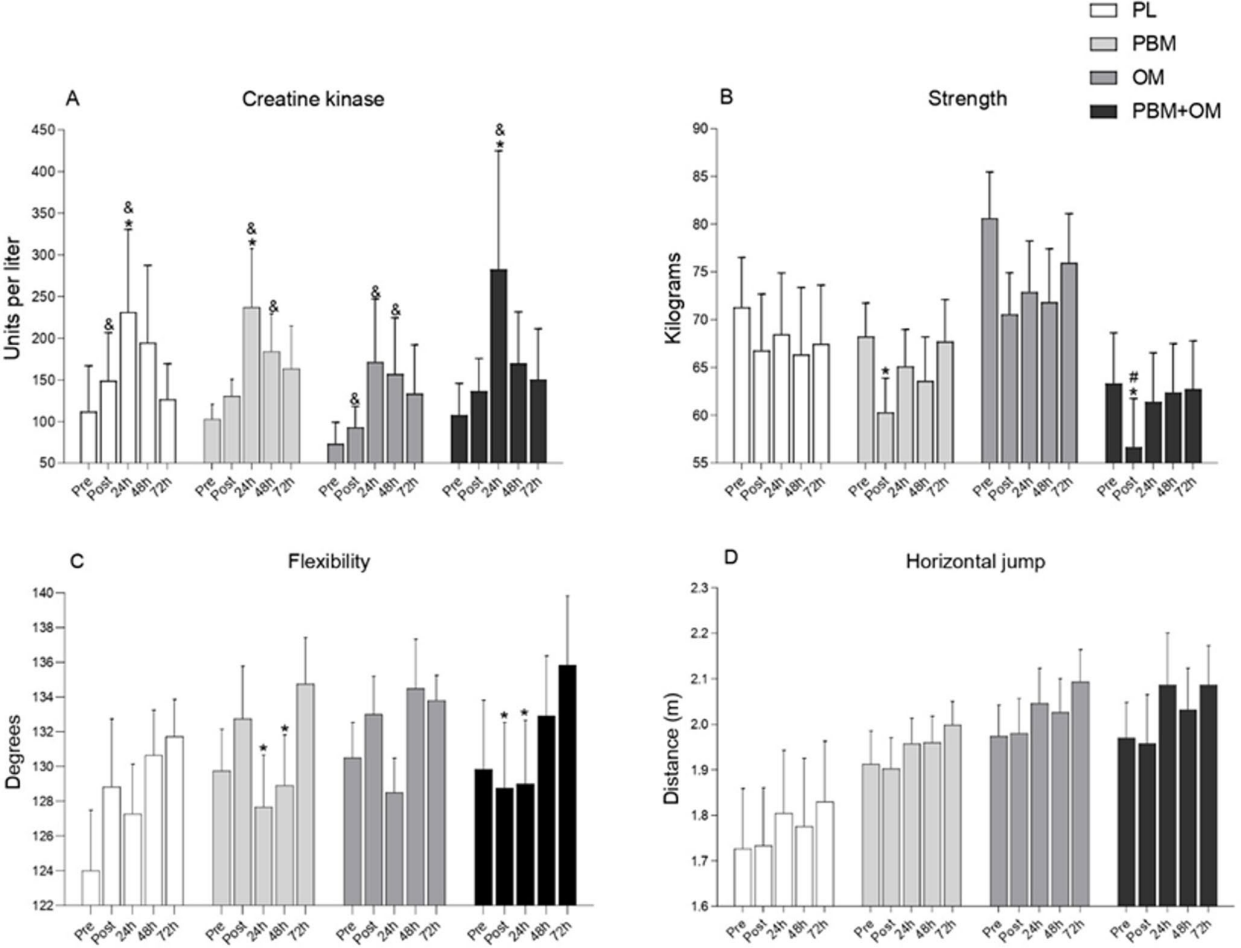
Effects of photobiomodulation and osteopathic manipulation on creatine kinase levels, strength, flexibility, and horizontal jump performance. PL: Placebo; PBM: Photobiomodulation; OM: Osteopathic Manipulation; PBM + OM: Photobiomodulation + Osteopathic Manipulation. &, different from pre-treatment in the same treatment; *, different from 72 h in the same treatment; #, different from 48 h in the same treatment. Minimum significance level of *p* < 0.05

This subsection presents the effects of the experimental protocol on serum CK levels, muscle strength, flexibility, and horizontal jump performance over a 72-hour follow-up. These reports show the recovery kinetics and potential effects of the treatments applied, both individually (PBM or OM) and combined (PBM + OM), in mitigating exercise-induced muscle damage (Fig. 3).

Regarding CK (Fig. 3A), the mixed-effects model analysis revealed a significant main effect for Time (F(1.381, 56.63) = 8.282, *p* = 0.002), but no significant main effect for Groups (F(3, 41) = 0.115, *p* = 0.950) or Time X Groups interaction (F(12, 164) = 0.302, *p* = 0.988). Nevertheless, the within-group analysis indicated that all cohorts showed a difference over time with medium effect sizes: PL [χ² (4) = 25.82, *p* = 0.0001, W = 0.587]; PBM [χ² (4) = 19.93, *p* = 0.0005, W = 0.415]; OM [χ² (4) = 26.43, *p* = 0.0001, W = 0.660]; and PBM + OM [χ² (4) = 23.09, *p* = 0.0001, W = 0.481]. In the PL and OM groups, an immediate increase in serum CK levels was observed after the EIMD protocol (PL: pre vs. post, *p* = 0.01; OM: pre vs. post, *p* = 0.02). In the PBM and PBM + OM groups, this increase occurred only after 24 h (PBM: pre vs. 24 h, *p* < 0.001; PBM + OM: pre vs. 24 h, *p* = 0.001). Notably, the combined PBM + OM group demonstrated a faster visual clearance of serum CK; while the other groups maintained elevated CK levels for a longer period, the PBM + OM group presented a CK peak at 24 h, with a significant drop at 72 h (*p* = 0.001).

Regarding strength (Fig. 3B), the mixed-effects model showed a significant main effect for Time (F(2.848, 116.2) = 6.236, *p* = 0.0007), with no significant main effects for Groups (F(3, 41) = 1.312, *p* = 0.283) or Time X Groups interaction (F(12, 164) = 0.563, *p* = 0.868). Within-group fluctuations over time presented large effect sizes across groups: PL [F(4, 40) = 1.475, *p* = 0.244, η_p_² = 0.253]; PBM [F(4, 44) = 1.807, *p* = 0.184, η_p_² = 0.141]; OM [F(4, 36) = 1.835, *p* = 0.168, η_p_² = 0.169]; and PBM + OM [F(4, 44) = 2.883, *p* = 0.060, η_p_² = 0.207]. A percentage reduction was observed immediately after the EIMD protocol: PL (6.3%), PBM (11.6%), OM (12.4%), and PBM + OM (10.5%); however, no between-group significance was reached. Exploratorily, the post hoc analysis revealed significant strength recovery post-exercise. In the PBM group, strength significantly increased from immediate post-exercise to 72 h (mean difference = 7.40; 95% CI: 0.02 to 14.78; *p* = 0.049). For the PBM + OM group, significant recovery was detected at 48 h (mean difference = 5.72; 95% CI: 0.33 to 11.12; *p* = 0.036) and 72 h (mean difference = 6.08; 95% CI: 1.85 to 10.32; *p* = 0.0051) compared to the immediate post-exercise time point. Consequently, only the PBM + OM group showed a numerical trend closer to baseline values after 48 h (−1.4%) and 72 h (−0.91%), while the PBM group also presented results close to baseline at 72 h (−0.76%).

Regarding flexibility (Fig. 3C), the mixed-effects model showed no significant main effects or interactions for Time (F(3.452, 141.5) = 10.04, *p* < 0.0001), Groups (F(3, 41) = 0.294, *p* = 0.829), or Time X Groups interaction (F(12, 164) = 1.386, *p* = 0.177). Within-group analysis showed large effect sizes across active cohorts: PL [F(4, 40) = 2.711, *p* = 0.067, η_p_² = 0.213]; PBM [F(4, 44) = 5.059, *p* = 0.004, η_p_² = 0.315]; OM [F(4, 36) = 3.315, *p* = 0.038, η_p_² = 0.269]; and PBM + OM [F(4, 44) = 3.888, *p* = 0.015, η_p_² = 0.261]. All groups presented an increase in degrees of knee flexion range of motion after 72 h: PL (7.7º), PBM (5.0º), OM (3.3º), and PBM + OM (6.0º). However, significant within-group temporal changes were exclusively confirmed for the active photobiomodulation cohorts. In the PBM group, knee flexion significantly increased at 72 h compared to 24 h (mean difference = 7.08º; 95% CI: 2.38º to 11.78º; *p* = 0.003) and 48 h (mean difference = 5.83º; 95% CI: 0.69º to 10.98º; *p* = 0.024). For the PBM + OM group, a significant gain in flexibility was observed at 72 h compared to immediate post-exercise (mean difference = 7.08º; 95% CI: 0.48º to 13.69º; *p* = 0.034) and 24 h (mean difference = 6.83º; 95% CI: 1.48º to 12.19º; *p* = 0.011). The PL group did not show statistically significant differences over time (*p* = 0.067).

In the horizontal jump test (Fig. 3D), the mixed-effects model indicated a significant main effect for Time (F(2.899, 118.9) = 9.522, *p* < 0.0001), but no main effect for Groups (F(3, 41) = 1.620, *p* = 0.199) or Time X Groups interaction (F(12, 164) = 0.214, *p* = 0.997). Over-time changes revealed large effect sizes according to the revised thresholds: PL [F(4, 40) = 1.571, *p* = 0.225, η_p_² = 0.135]; PBM [F(4, 44) = 1.818, *p* = 0.180, η_p_² = 0.141]; OM [F(4, 36) = 3.891, *p* = 0.014, η_p_² = 0.302]; and PBM + OM [F(4, 44) = 3.732, *p* = 0.029, η_p_² = 0.253]. All groups presented descriptive improvements in jump distance performance after 72 h: PL (10.4 cm), PBM (8.5 cm), OM (11.9 cm), and PBM + OM (11.7 cm). The PBM + OM group exhibited an exploratory, non-significant numerical increase of 11.7 cm at 24 h post-intervention.

### Correlation between muscle damage and physical performance

This subsection examines the potential relationship between serum CK levels and the recovery of muscle strength, flexibility, and horizontal jump performance, to determine if variations in biochemical markers of muscle damage directly correlate with changes in physical functional tests at each time point.

The Spearman’s correlation test indicated that, in general, serum CK levels did not show significant correlations with the physical variables analyzed (strength, flexibility, and muscle power) across the pre, post, and 24, 48, and 72-hour post intervals (*p* > 0.05). As shown in Table 4, in this specific population and experimental protocol, the recovery of physical performance appears to follow a kinetic profile independent of the absolute magnitude of the biochemical damage marker.

**Table 4:** Correlation between CK and strength, flexibility, and the horizontal jump test in relation to time

Variables	Groups			
	PL	PBM	OM	PBM + OM
	*r*; *p* (CI 95%)	*r*; *p* (CI 95%)	*r*; *p* (CI 95%)	*r*; *p* (CI 95%)
CK pre x Strength pre	0.09; 0.77 (−0.52 to 0.64)	−0.30; 0.34 (−0.75 to 0.34)	−0.26; 0.40 (0.73 to 0.38)	0.10; 0.74 (− 0.51 to 0.65)
CK post x Strength post	−0.11; 0.72 (−0.65 to 0.50)	0.26; 0.40 (−0.38 to 0.73)	−0.10; 0.74 (−0.65 to 0.51)	0.20; 0.51 (−0.43 to 0.70)
CK 24 h x Strength 24 h	0.24; 0.44 (−0.39 to 0.72)	−0.13; 0.66 (−0.67 to 0.48)	0.11; 0.72 (−0.50 to 0.65)	0.43; 0.15 (−0.20 to 0.81)
CK 48 h x Strength 48 h	0.18; 0.59 (−0.48 to 0.71)	−0.00; 0.98 (−0.59 to 0.58)	0.14; 0.66 (−0.51 to 0.69)	0.16; 0.60 (−0.46 to 0.68)
CK 72 h x Strength 72 h	0.09; 0.77 (0.52 to 0.64)	0.16; 0.61(−0.47 to 0.68)	0.07; 0.83 (−0.56 to 0.65)	0.23; 0.45 (−0.40 to 0.72)
CK pre x Flexibility pre	−0.11; 0.71 (−0.65 to 0.50)	−0.29; 0.35 (−0.74 to 0.35)	−0.13; 0.67 (−0.67 to 0.48)	−0.48; 0.11 (−0.83 to 0.14)
CK post x Flexibility post	−0.64; 0.02 (−0.89 to −0.09)	−0.47; 0.11 (−0.82 to 0.15)	−0.12; 0.69 (−0.66 to 0.49)	−0.54; 0.06 (−0.85 to 0.06)
CK 24 h x Flexibility 24 h	0.14; 0.64 (−0.48 to 0.67)	−0.03; 0.91 (−0.60 to 0.56)	0.56; 0.06 (−0.03 to 0.86)	−0.37; 0.22 (−0.78 to 0.26)
CK 48 h x Flexibility 48 h	0.02; 0.93 (−0.59 to 0.62)	−0.05; 0.87 (−0.62 to 0.55)	−0.19; 0.55 (−0.72 to 0.47)	−0.49; 0.10 (−0.83 to 0.12)
CK 72 h x Flexibility 72 h	−0.07; 0.80 (−0.63 to 0.53)	−0.30; 0.32 (−0.75 to 0.33)	0.33; 0.31 (−0.34 to 0.78)	−0.28; 0.36 (−0.74 to 0.35)
CK pre x Horiz. jump pre	0.20; 0.52 (−0.43 to 0.70)	0.12; 0.70 (−0.50 to 0.66)	−0.59; 0.03 (−0.87 to −0.01)	0.34; 0.27 (−0.30 to 0.77)
CK post x Horiz. jump post	0.21; 0.49 (−0.42 to 0.71)	−0.07; 0.81 (−0.63 to 0.53)	−0.55; 0.06 (−0.86 to 0.04)	0.24; 0.44 (−0.39 to 0.72)
CK 24 h x Horiz. jump 24 h	0.30; 0.32 (−0.33 to 0.75)	−0.18; 0.57 (−0.69 to 0.45)	−0.49; 0.10 (−0.83 to 0.12)	0.42; 0.16 (−0.21 to 0.81)
CK 48 h x Horiz. jump 48 h	0.21; 0.51 (−0.45 to 0.73)	−0.01; 0.97 (−0.59 to 0.57)	−0.47; 0.14 (−0.84 to 0.19)	0.36; 0.24 (−0.28 to 0.78)
CK 72 h x Horiz. jump 72 h	0.04; 0.87 (−0.55 to 0.61)	0.22; 0.49 (−0.42 to 0.71)	−0.31; 0.33 (−0.77 to 0.36)	0.53; 0.07 (−0.07 to 0.85)

*CI95%* 95% confidence interval, *CK* creatine kinase, *h* hours, *p* p-value, *PL* placebo, *PBM* photobiomodulation, *OM* osteopathic manipulation, *PBM + OM* Photobiomodulation + Osteopathic Manipulation, *r* Spearman correlation test, Minimum significance level of *p* < 0.05

## Discussion

The current study explored the combined effects of PBM and OM on the physical performance of physically active young adults undergoing EIMD. Analysis of the results showed an immediate increase in CK in the PL and OM groups, a behavior not identified in the PBM and PBM + OM groups. Furthermore, the rapid reduction in CK in the PBM + OM group suggests that integration of the two approaches may exert a possible protective effect on muscle and modulate peripheral inflammation. When analyzed through the lens of effect size, these findings represent a moderate to high practical magnitude, suggesting that the biochemical profile offered by the combined protocol might assist in mitigating excessive muscle leakage of intracellular enzymes. However, given the high variability of this biomarker, these biochemical changes should not be directly equated with clinical or functional recovery. This effect of PBM on CK levels was also observed in a meta-analysis study. When analyzing 14 studies involving healthy individuals, it was found that PBM applied both before and after exercise reduced serum CK levels compared to placebo/control groups [27]. On the other hand, regarding the effects of OM on serum CK levels, the literature lacks studies related to EIMD or any other form of exercise.

Clinical trials with a similar EIMD protocol, participant age, and PBM application points to those used in the present study, demonstrated a reduction in CK with infrared LED (940 nm), 72 h after exercise [3], and with a laser cluster (905 nm) and LEDs (875 and 670 nm) at doses of 10, 30, and 50 J, between 1 and 96 h after exercise [28]. The parameters used by the authors differ from the present study (650 nm, 9 J per point and 144 J total), therefore, different parameters may to some extent modify the post-EIMD CK dynamics. The positive effects of PBM have been attributed to the modulation of mitochondrial activity and the increase in ATP availability, favoring muscle recovery, improved strength and endurance, and reduced fatigue [10, 29]. From a functional perspective, this enhanced bioenergetic state is essential for maintaining movement efficiency and neuromuscular performance during activities of daily living and explosive motor tasks. These activities rely on key functional patterns, such as force production, eccentric control, coordination, and proprioception, which are often compromised following EIMD [2, 4].

Regarding the effects of OM on serum CK levels, one study showed that neither cervical nor thoracic OM caused alterations in the levels of this enzyme. The same study showed that OM also did not interfere with serum levels of lactate dehydrogenase and C-reactive protein [30]. Although the results on the effects of OM on peripheral mechanisms that regulate the inflammatory response still present inconsistencies and need to be interpreted with caution, spinal manipulation (SM) has been associated with potential peripheral anti-inflammatory effects, a reduction in pro-inflammatory mediators and reactive oxygen species, and improvement in nerve function [31–33]. A crucial mechanism identified in the current study involves the modulation of the central nervous system (CNS). Manual stimulus applied during OM triggers sensory afferents through muscle spindles and Golgi tendon organs. These afferent signals reach the CNS and may increase motor neuron excitability and recruitment (efferent drive), potentially counteracting the inhibitory effects typically associated with muscle damage and contributing to improvements in mechanical output [10, 16, 31].

The dynamics observed in CK behavior in the current study resemble those described by Bridgeman et al., who observed a peak 24 h after EIMD and a decline after 48 h [34]. While a decline in serum CK following eccentric exercise is expected as part of the natural recovery curve, the magnitude of the reduction observed at 72 h specifically in the PBM + OM group was more pronounced than in the placebo group. This indicates that the combined intervention may have accelerated the physiological recovery process beyond the natural timeline of biochemical clearance. The authors did not find significant impairment in jump and strength performance; therefore, the peak in CK does not necessarily translate into impaired muscle performance [34]. This lack of correlation between CK and functional performance (strength and jump) was also observed in our results, which is consistent with the well-documented ‘dissociation’ between biochemical markers and mechanical output [4, 35]. Serum CK reflects sarcolemma permeability and muscle fiber damage, whereas physical performance is a multifactorial process involving neural drive, excitation-contraction coupling, and metabolic efficiency [36]. Therefore, while CK is a sensitive marker of systemic muscle stress, it may not directly predict immediate functional capacity in healthy individuals. Similarly, regarding the baseline characteristics presented in Table 2, a descriptive numerical variance was observed in the metabolic age of the PL group (35.9 ± 7.3 years) compared to the active groups (~ 21 years). Although the global analysis confirmed no significant differences between cohorts (*p* = 0.43), this point-in-time discrepancy is acknowledged strictly as a statistical artifact of the sample distribution combined with the high standard deviation and the inherent variability of bioimpedance predictive equations. Because chronological age, body mass, and baseline functional capacities were fully homogeneous across all groups, this numerical variation was not considered a confounding factor and was not subjected to further mechanistic interpretation. Our results descriptively suggest that the PBM + OM group presented a trend toward strength recovery, as despite not preventing the acute drop, it restored strength to values close to baseline 48 h after the intervention. Nevertheless, because no statistically significant between-group differences were detected, this observation must be interpreted with caution and strictly as an exploratory trend. A meta-analysis on PBM demonstrated improved muscle performance and reduced levels of biomarkers associated with EIMD [37]. Furthermore, a review on spinal manipulation showed gains in isometric strength, especially when compared to no intervention or a sham manipulation [13].

Serighelli et al., applied an infrared LED cluster (830 nm) and three 90 nm amber LEDs to the quadriceps and triceps surae muscles and concluded that PBM alone does not improve quadriceps strength and endurance results in healthy individuals [38]. Rossato et al., using different doses and distinct PBM protocols, demonstrated that doses of 135, 270, and 540 J were able to produce the same total work with less muscle fatigue [39]. That is, different PBM parameters (type, dose, and duration) can lead to different results, as there is an optimal dose to maximize biological effects, and stimuli with very low or very high doses may not produce biological effects [40].

Clinical studies in athletes have observed increases in lower and upper limb muscle strength immediately after OM [14, 41]. Grindstaff et al., observed an immediate increase in strength and activation of the quadriceps muscle in healthy individuals, but strength decreased over an hour, with this effect being associated with muscle fatigue [42]. On the other hand, in Sanders’ et al. study all strength measures were equivalent between the OM group and PL group, the hypothesis is that healthy individuals do not exhibit muscle inhibition [43]. Therefore, clinical conditions can play a relevant role in physiological muscle responses, and individuals free of symptoms and pathologies may have limited capacity to produce significant changes after a single muscle alignment maneuver [44].

The progressive increase in range of motion observed 72 h post-exercise may reflect a combination of neural adaptations, transient structural changes, and connective tissue responses, associated with the tissue repair process. Following the initial eccentric stimulus, an adaptive process occurs, involving both the neural system and connective tissue, resulting in less stiffness and greater muscle stretching capacity in subsequent sessions [45]. Collagen remodeling after muscle damage also contributes to and may explain the improvement in flexibility observed over the days [46]. The reduction in stiffness detected after eccentric exercises appears to be associated with structural recovery, and neural and sensory modulation of the muscle [47]. These mechanisms lead us to believe that even a single session of eccentric exercises is capable of triggering protective and functional adaptation responses in the muscle. Notably, no negative effects or adverse clinical events, such as increased pain or discomfort, were reported by participants, confirming the safety of these non-invasive interventions.

The results of the current study demonstrated an improvement, in centimeters, in horizontal jump performance in all groups. Notably, the PBM + OM group showed improvement within the first 24 h. PBM with LEDs positively influenced plyometric jump performance, promoting a significant increase in squat jumps (SJ) 24 h (630 nm group) and 48 h after exercise (940 nm group) [3]. In basketball players, a trend to improved vertical jump and reach performance was observed after a standardized OM protocol [18]. These findings suggest that PBM associated with OM may favor movement efficiency, which is essential for jumping performance.

The superior trends observed in the PBM and OM combination appear to integrate complementary neurophysiological mechanisms; PBM exerts an ergogenic effect, modulating inflammation and energy metabolism, and promoting greater efficiency in the muscle response, while OM promotes the discharge of neuromuscular spindles, modulating muscle tone and activation [9, 12, 16]. Thus, the association between PBM and OM may offer a complementary approach to muscle recovery, combining hypothesized bioenergetic effects with mechanical and neural adjustments. However, since the functional outcomes in our study were modest and lacked robust between-group statistical significance, the clinical magnitude of this combination remains to be confirmed. The clinical implications of these findings are direct and relevant for sports rehabilitation. The combination of PBM and OM may provide a potent, drug-free strategy for physical therapists, to support the recovery of muscle function, potentially addressing both the cellular damage and neuromuscular inhibition associated with intense physical exertion. Evidence suggests that photobiomodulation can enhance muscle recovery, reduce fatigue, and improve performance outcomes, while osteopathic manipulative interventions may influence neuromuscular function through modulation of the CNS and motor control [7, 9, 14, 16, 48]. Consequently, the clinical applicability of these outcomes must be restricted strictly to the evaluated cohort: physically active young men undergoing acute plyometric stress. Given the specific physiological and metabolic characteristics of this sample, these findings should not be extrapolated to competitive elite athletes, female populations, older adults, injured patients, or long-term clinical rehabilitation settings without supporting empirical data.

The current study presents limitations, as the sample consisted exclusively of young, physically active males, which may limit the generalization of the findings to other populations. Furthermore, the interventions were only applied once before the EIMD protocol; therefore, the exposure may not have been sufficient to generate greater adaptations in physical performance. Regarding the photobiomodulation parameters, while the 650 nm wavelength yielded positive results in our combined protocol with Osteopathic Manipulation, it is important to acknowledge that red light has more limited tissue penetration compared to near-infrared (NIR) wavelengths. In this study, the choice of 650 nm was intended to target superficial muscle fibers, localized dermal microcirculation, and systemic signaling pathways rather than deep tissue layers. Nevertheless, the use of deeper-penetrating NIR light (e.g., 800–950 nm) might potentially enhance these recovery effects in deep muscle tissues, providing a clear rationale for future comparative studies. Future research should also focus on optimizing PBM protocols, including a more comprehensive osteopathic approach, elucidating the mechanisms of OM, and exploring new applications in broader aspects of PBM, in order to determine when the use of these combined therapies is most effective in preventing EIMD. Finally, the success of the participant blinding protocol was not formally assessed via a blinding index questionnaire, which should be considered a limitation.

## Conclusion

The results of the current study suggest that while both isolated PBM and its combination with OM influence muscle recovery parameters, no statistically significant superior effects were found between the experimental groups. Numerically, the PBM + OM group demonstrated a favorable descriptive recovery profile, particularly regarding the acceleration of CK clearance curves and within-group restoration of physical parameters. Regarding muscle strength, since the model did not reach statistical significance, the intervention cannot be claimed to favor strength recovery; however, it presented an exploratory non-significant trend close to baseline values. These findings suggest a potential, though modest, additive or complementary benefit of the combined therapies in mitigating certain aspects of exercise-induced muscle damage. Ultimately, PBM + OM may have contributed to improved CK kinetics and selected functional outcomes; however, strength recovery should be interpreted strictly as a non-significant and exploratory trend.

## Data Availability

The data presented in this study are available on request from the corresponding author. The data are not publicly available.

## Declarations

### Clinical trial number

After approval by the committee, the project was registered with the Brazilian Registry of Clinical Trials (ReBEC) under number RBR-8kdv3pt (https://ensaiosclinicos.gov.br/rg/RBR-8kdv3pt), registered on July 15, 2024.
